# Botulinum neurotoxin type A modulates vesicular release of glutamate from satellite glial cells

**DOI:** 10.1111/jcmm.12562

**Published:** 2015-03-05

**Authors:** Larissa Bittencourt da Silva, Jeppe Nørgaard Poulsen, Lars Arendt-Nielsen, Parisa Gazerani

**Affiliations:** a Center for Sensory - Motor Interaction (SMI), Department of Health Science and Technology, Faculty of Medicine, Aalborg UniversityAalborg East, Denmark; b Laboratory for Cancer Biology, Biomedicine, Department of Health Science and Technology, Faculty of Medicine, Aalborg UniversityAalborg East, Denmark

**Keywords:** botulinum neurotoxin type A, pain, glutamate, vesicular release, trigeminal ganglion, satellite glial cells, ionomycin, SNAP-25, SNAP-23

## Abstract

This study investigated the presence of cell membrane docking proteins synaptosomal-associated protein, 25 and 23 kD (SNAP-25 and SNAP-23) in satellite glial cells (SGCs) of rat trigeminal ganglion; whether cultured SGCs would release glutamate in a time- and calcium-dependent manner following calcium-ionophore ionomycin stimulation; and if botulinum neurotoxin type A (BoNTA), in a dose-dependent manner, could block or decrease vesicular release of glutamate. SGCs were isolated from the trigeminal ganglia (TG) of adult Wistar rats and cultured for 7 days. The presence of SNAPs in TG sections and isolated SGCs were investigated using immunohistochemistry and immunocytochemistry, respectively. SGCs were stimulated with ionomycin (5 μM for 4, 8, 12 and 30 min.) to release glutamate. SGCs were then pre-incubated with BoNTA (24 hrs with 0.1, 1, 10 and 100 pM) to investigate if BoNTA could potentially block ionomycin-stimulated glutamate release. Glutamate concentrations were measured by ELISA. SNAP-25 and SNAP-23 were present in SGCs in TG sections and in cultured SGCs. Ionomycin significantly increased glutamate release from cultured SGCs 30 min. following the treatment (*P* < 0.001). BoNTA (100 pM) significantly decreased glutamate release (*P* < 0.01). Results from this study demonstrated that SGCs, when stimulated with ionomycin, released glutamate that was inhibited by BoNTA, possibly through cleavage of SNAP-25 and/or SNAP-23. These novel findings demonstrate the existence of vesicular glutamate release from SGCs, which could potentially play a role in the trigeminal sensory transmission. In addition, interaction of BoNTA with non-neuronal cells at the level of TG suggests a potential analgesic mechanism of action of BoNTA.

## Introduction

The trigeminovascular system, which consists of neurons, blood vessels and non-neuronal cells (glial cells), is an important player in craniofacial nociception 1,2. Dysfunction of this system is considered a potential mechanism underlying the development and/or maintenance of painful conditions such as migraine headaches 3. Trigeminal ganglia (TG) contain the cell bodies of pseudo-unipolar neurons, which are surrounded by non-neuronal satellite glial cells (SGCs). Neuron-glia interaction within the sensory ganglia is based on this unique functional structure 4, where only an average distance of 20 nm exists between SGCs and their associated neurons allowing exchange of chemicals between the two cell types 5. Neuron-glia interaction under physiological conditions is crucial for optimal functioning 6, and it is gradually becoming evident that under pathological pain conditions, this interaction is disturbed 7,8.

One of the substances proposed to contribute to neuron-glia cross talk is excitatory amino acid glutamate, and disruption of glutamate metabolism by SGCs could lead to increased level of glutamate within the TG 9,10. We have recently demonstrated that trigeminal SGCs in rats contain glutamate and express excitatory amino acid transporters (EAAT) and that the cultured SGCs can be stimulated with potassium chloride (10 mM) to release glutamate 11. We also demonstrated that intraganglionic injection of glutamate *in vivo* evoked afferent discharges, which were increased by the EAAT1 and EAAT2 blocker, (3S)-3-[[3-[[4-(trifluoromethyl)benzoyl]amino]phenyl]methoxy]-l-aspartic acid. Elevated levels of glutamate within the TG also lowered the mechanical threshold of the masticatory muscle afferent fibres; an effect that was attenuated by the *N*-methyl-d-aspartate receptor antagonist, 2-amino-5-phosphonovalerate 11. However, this study did not determine whether SGCs can release glutamate through calcium-dependent vesicular process.

Vesicular release of glutamate occurs through binding of the vesicle to the cell membrane SNARE docking complexes that contain the synaptosomal-associated protein of 25 kD (SNAP-25) and/or its analogue, SNAP-23 12,13. Signalling between astrocytes and neurons *via* glutamate has been extensively studied 12,14. Under experimental conditions, calcium-dependent release of glutamate can be stimulated by different agents such as bradykinin and calcium-ionophore, ionomycin 14,15. Botulinum neurotoxin type A (BoNTA) is known to inhibit vesicular release of glutamate (and other neurotransmitters) by cleaving neuronal SNAP-25 16–18. BoNTA has been shown to inhibit glutamate release by non-neuronal cells of the central nervous system, such as astrocytes and microglia 13,14; but, it is unclear if peripheral glia, SGCs, would also express SNAPs and respond to BoNTA in the same way as the central glia.

Hence, the present study was designed to investigate (*i*) qualitative presence of SNAP-25 and SNAP-23 in trigeminal SGCs; (*ii*) whether the cultured trigeminal SGCs would release glutamate in a time- and calcium-dependent manner following stimulation by ionomycin; and (*iii*) if BoNTA would block or decrease the vesicular release of glutamate from these cells. Results from this study would not only shed light on possible role(s) of peripheral non-neuronal cells (SGCs) in trigeminal sensory transmission, but also advance our understanding about mechanism(s) of analgesic action of BoNTA at the level of sensory ganglia.

## Materials and methods

### Animals

The study was performed with 20 adult male Wistar rats (2–4 months old) provided through the Animal Research Facility, Department of Pathology, Aalborg University Hospital, Denmark. Animals were housed in groups of three rats per cage, in temperature-controlled rooms, on a 12 hrs light/dark cycle with access to food and water *ad libitum*. All animals were deeply anesthetized and then killed prior to all experimental procedures. All procedures were conducted according to ethical guidelines defined by the Danish Animal Experiments Inspectorate in accordance with the guidelines set by the International Association for the Study of Pain for use of laboratory animals in medical research.

### Immunohistochemistry

Animals were deeply anesthetized with a mixture of hypnorm (Vm21757/4000; Vetapharma, Leeds, West Yorkshire, UK), midazolam (Hameln Pharmaceuticals, Gloucester, UK), and isotonic saline (25%, 25%, 50% v/v. 0.3 ml/100 g) prior to transcardial perfusion with isotonic saline followed by 10% formalin buffer solution (BAF-0010-03A; CellPath, Newtown, Wales, UK). The TG were removed and placed in 10% formalin buffer solution overnight at 4°C. The next day the ganglia were washed in PBS (14190-094; Gibco Life Technologies, Invitrogen, CA, Waltham, USA) and placed in 20% sucrose solution for 48 hrs at 4°C. The tissue was then placed in 40% sucrose solution overnight at 4°C for cryoprotection. Afterwards, the ganglia were embedded in Tissue-Tek (4583; Sakura Finetek, Alphen aan den Rijn, The Netherlands) and frozen separately in a cryo-mould (4565; Sakura Finetek). Ganglia were sectioned with a cryostat (MICROM; Thermo Fisher Scientific, Waltham, Germany; 10 μm). Sections were mounted on poly-l-lysine coated glass slides (1510.1260; Hounisen Laboratorieudstyr, Glostrup, Denmark) and incubated in 5% bovine serum albumin (BSA; EQBAH62; Europa Bioproducts, Cambridge, UK) supplemented with 0.2% Triton X100 for 1 hr at room temperature (RT) before they were washed with PBS. Primary antibodies against glutamine synthetase (GS: G2781; Sigma-Aldrich, St. Louis, MO, USA; 1:10,000; rabbit), and either SNAP-23 (ab166808; Abcam, Cambridge, UK; 1:100; goat) or SNAP-25 (ab136493; Abcam; 1:100; goat) in 1% BSA solution were added and incubated at 4°C overnight. Slides were washed in PBS and incubated with secondary antibody Donkey anti-rabbit Alexa Fluor 555 conjugate 1:500 (ab150074; Abcam) and Donkey anti-goat Alexa Fluor 488 conjugate 1:500 (ab150129; Abcam), in 1% BSA, for 1 hr at RT. The nuclear dye Hoechst was added for 10 min. at RT, in a 1:2000 dilution in 1% BSA, before the slides were washed in PBS and milli-Q water. Subsequently, the slides were mounted with glass cover slips (Fluorescent mounting medium; S3023; Dako, Denmark) and images were obtained using a Nikon microscope (Az100; Nikon, Tokyo, Japan) equipped with a fluorescent illuminator (L200/D; Prior Scientific, Rockland, MA, USA) and a digital camera (DS-Vi1; Nikon) connected to a personal computer. Image J (public domain software) was used for further analysis.

### Primary trigeminal ganglion cell cultures

Animals were deeply anesthetized with a mixture of hypnorm (Vm21757/4000; Vetapharma), midazolam (Hameln Pharmaceuticals), and isotonic saline (25%, 25%, 50% v/v. 0.3 ml/100 g) and killed by cervical dislocation. The TG were removed, cut into smaller sections with microscissors, placed in 5 mg/ml collagenase suspension (C9891; Sigma-Aldrich) in Ham’s F12 growth medium (21765-029; Gibco Life Technologies, Invitrogen) supplemented with 1% penicillin/streptomycin (15140; Gibco Life Technologies, Invitrogen) and incubated (37°C) for 15 min. Following incubation, the collagenase solution was centrifuged (280 g) for 5 min. The pellet was re-suspended in 1 ml 0.125% trypsin (15090-046; Gibco Life Technologies, Invitrogen) and placed in the incubator for 5–10 min. prior centrifugation (5 min./280 g). The sectioned and enzymatically digested ganglia were then mechanically dissociated into a homogenous solution by repeated pipetting. The cell solution was added to uncoated culture flasks before being placed in an incubator (37°C – 95% air/ 5% CO_2_). The growth medium was changed after 3 and 24 hrs, and then continuously every 2–3 days prior to the experimental procedure.

### Immunocytochemistry

Cells were washed with PBS prior to fixation in 10% formalin buffer solution (BAF-0010-03A; CellPath) for 15–20 min. Afterwards, 0.2% Triton X-100 (X100; Sigma-Aldrich) was added for 15 min. The cells were washed twice in PBS before incubated in 5% BSA for 1 hr at RT. Primary antibodies against GS (G2781; Sigma-Aldrich; 1:4000; rabbit), and either SNAP-23 (ab166808; Abcam; 1:200; goat) or SNAP-25 (ab136493; Abcam; 1:200; goat) were diluted in 1% BSA solution and incubated overnight at 4°C. The following day, cells were washed prior to incubation with secondary antibodies donkey anti-rabbit Alexa Fluor® 555 conjugate 1:500 (ab150074; Abcam) and donkey anti-goat Alexa Fluor® 488 conjugate 1:500 (ab150129; Abcam), in a 1% BSA solution, for 1.5 hrs in the dark. Subsequently, the cells were incubated with Hoechst for 10 min. at 4°C, in a concentration of 1:2000. Afterwards, the cells were washed in PBS and then milli-Q water before mounted with glass cover slips (Fluorescent mounting medium; S3023; Dako). Images were obtained using a Nikon microscope (Az100; Nikon) equipped with a fluorescent illuminator (L200/D; Prior Scientific) and a digital camera (DS-Vi1; Nikon) connected to a personal computer. Image J (public domain software) was used for further analysis.

### Glutamate release evoked by ionomycin

Once cell confluence attained ≈90%, the supplemented growth medium was washed off and replaced with the glutamate-free DMEM (A1515401; Invitrogen) and left in the incubator overnight. The DMEM was replaced by fresh DMEM for control or treatment medium consisting of 5 μM ionomycin (I3909; Sigma-Aldrich) in DMEM. The concentration of ionomycin was chosen based on a previous work 15. Samples were collected from control and treatment culture medium after 4, 8, 12, and 30 min. The time-points were selected based on the previous work showing a quick release of glutamate within minutes following treatment with ionomycin 15. Cytotoxic potential of ionomycin (in terms of concentration and time of exposure) has already been reported,* where ionomycin at high concentration (100 μM) produces pronounced morphological changes after only 4 hrs. Maximal cell death at high concentration occurs within 10–20 hrs after the treatment. Although the cells tested in the past were from a different origin, it seems unlikely that the time and concentration of ionomycin selected for this study would cause any cytotoxic effect on trigeminal SGCs.

The glutamate concentrations in the collected samples were determined using a competitive immunoassay for quantitative determination of glutamate in biological samples (BA E-2300; Labor Diagnostika Nord, Nordhorn, Germany).

### Effect of BoNTA on ionomycin-evoked glutamate release from cultured SGCs

Once cell confluence attained ≈90%, the supplemented growth medium was washed off and replaced with the glutamate-free DMEM (A1515401; Invitrogen) and left in the incubator overnight. The DMEM was aspirated and replaced with pre-treatment medium containing 0.1, 1, 10 and 100 pM of BoNTA (Botox®, Allegan, CA, USA) in DMEM or with DMEM alone, and placed in the incubator (37°C) for 24 hrs. The calculations from Units to pM were done following the information obtained from the producer; each of the 200 U vials of Botox® represents 9.6 ng of 900 kD toxin. Each vial was then dissolved in 0.1 ml of medium. A total of 20 vials were used to reach a stock solution of 100 pM. The subsequent concentrations were reached with serial dilutions of the stock solution.

The concentrations of BoNTA and pre-treatment timing were chosen based on the previous publications 19,20. It has also been reported that 1.6 and 3.1 units of BoNTA are within the therapeutic concentrations, which have been used to study direct inhibitory effect of BoNTA on release of calcitonin gene-related peptide in primary cultures of rat TG 21.

After 24 hrs, pre-treatment medium was replaced by 0.75% dimethyl sulfoxide in DMEM (control), or a mixture of 50 μM BAPTA (1,2-bis(o-aminophenoxy)ethane-*N*,*N*,*N*′,*N*′-tetraacetic acid; A4926; Sigma-Aldrich), which selectively chelates intracellular calcium that is essential for glutamate release used here as a positive control, plus 5 μM ionomycin in DMEM, or 5 μM ionomycin alone in DMEM. The concentration of BAPTA was chosen based on a previous work 22. Samples of the cultured medium were collected after 30 min. of incubation. Glutamate concentrations in the collected samples were determined using a competitive immunoassay for quantitative determination of glutamate in biological samples (BA E-2300; Labor Diagnostika Nord).

### Statistical analysis

The effects of BoNTA on ionomycin-evoked glutamate release were analysed with repeated measures ANOVA (RM ANOVA; repeated factor: time). For the remaining data, one-way ANOVA was used. The Bonferroni test was used for *post hoc* comparison. All statistical tests were carried out by IBM (Armonk, New York, US). SPSS, version 20. Data are presented as mean ± SEM. *P* < 0.05 was considered statistically significant.

## Results

### Localization of SNAP-25 and SNAP-23 in trigeminal SGCs

**Figure 1:**
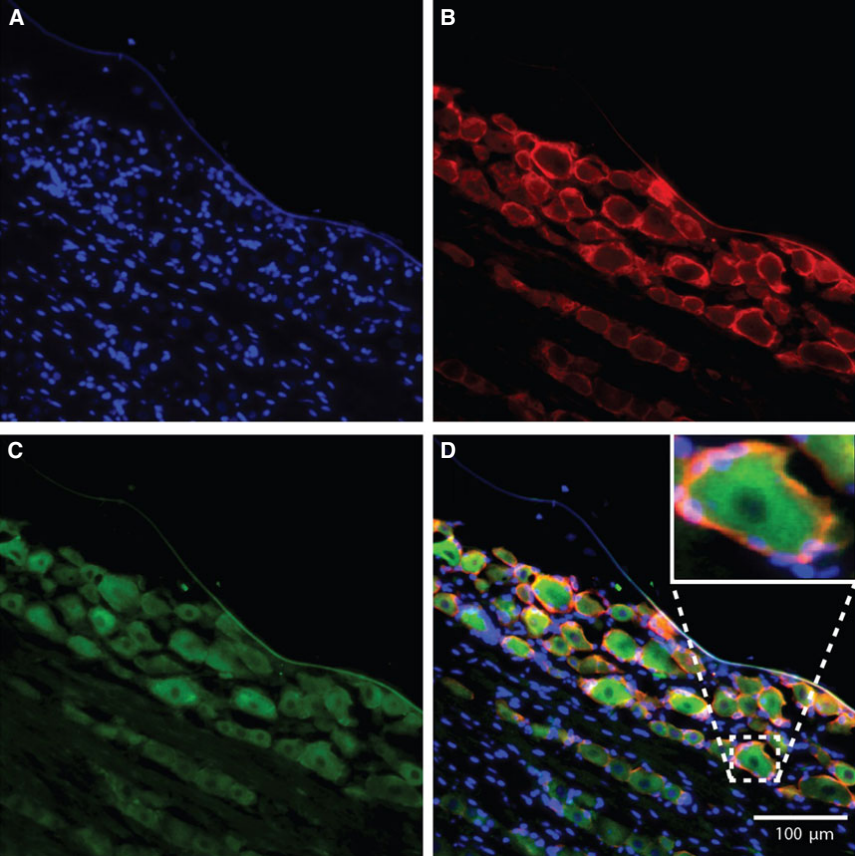
Localization of SNAP-25 in trigeminal satellite glial cells in rat. The blue colour represents the nuclear stain Hoechst, which stains the nuclei of each cell (A). SNAP-25 positive satellite glial cells were identified by double labelling with antibodies against glutamine synthetase, a specific satellite glial cell marker in ganglia (Red: Alexa fluor® 555) (B) and SNAP-25 (Green: Alexa fluor® 488) (C). On the merged image (D) overlapping of the two colours indicates SNAP-25 positive SGCs. The enhanced area on image (D) shows an example of such co-localization of satellite glial cells enveloping a neuron.

**Figure 2:**
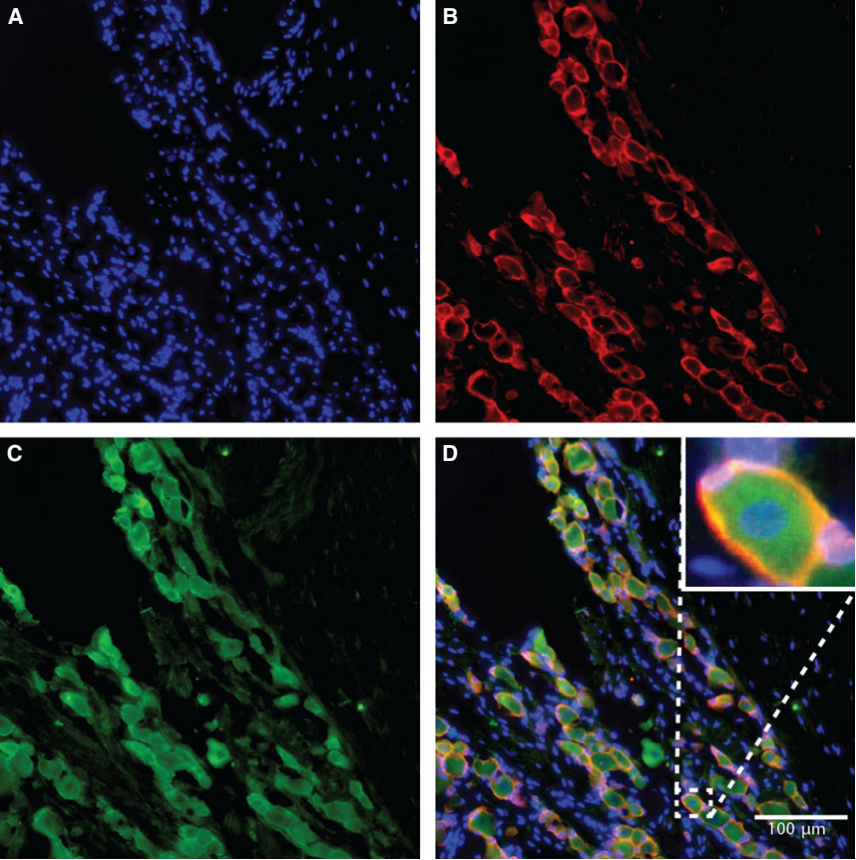
Localization of SNAP-23 in trigeminal satellite glial cells in rat. The blue colour on image (A) represents the nuclear stain Hoechst and stains the nuclei of each cell. SNAP23 positive satellite glial cells were identified by double labelling with antibodies against glutamine synthetase (Red: Alexa fluor® 555), a specific Satellite glial cell marker in ganglia (B) and SNAP-23 (Green: Alexa fluor® 488) (C). On the merged image (D) overlapping of the two colours indicates SNAP23 positive SGCs. The enhanced area on image (D) shows an example of such co-localization of satellite glial cells enveloping a neuron.

**Figure 3:**
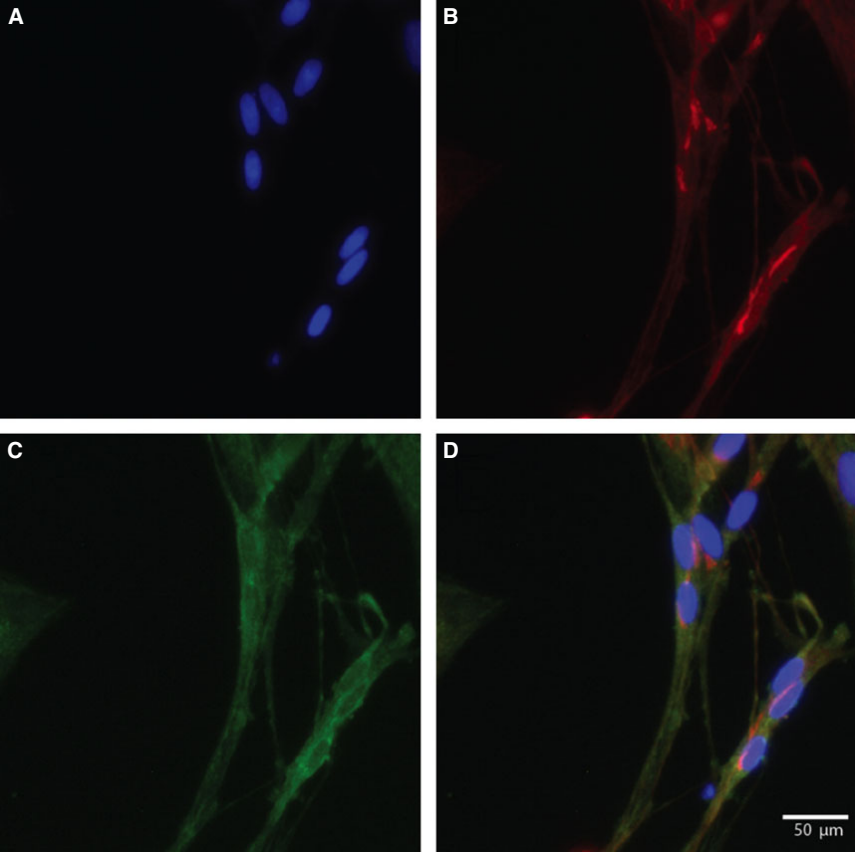
Immunocytochemistry of 7-day-old primary cultures of satellite glial cells from rat trigeminal ganglia. Image (A), shows the nuclei of the satellite glial cell stained with the nuclear stain Hoechst. The cultures are double labelled with antibodies against glutamine synthetase (Red: Alexa fluor® 555) (B) and SNAP-25 (Green: Alexa fluor® 488) (C). The merged image (D) is an overlay of all channels and this image shows the co-localization of glutamine synthetase and SNAP-25 in cultured rat trigeminal satellite glial cells. Virtually all SGCs seem SNAP-25 positive.

**Figure 4:**
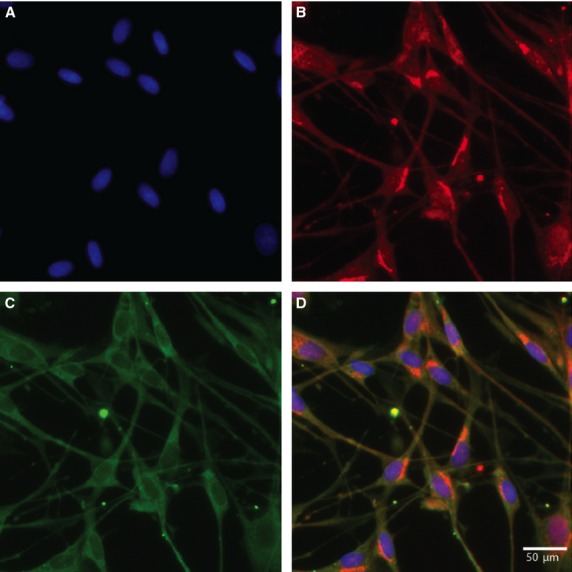
Immunocytochemistry of 7-day-old primary cultures of satellite glial cells from rat trigeminal ganglia. Image (A), shows the nuclei of the satellite glial cell stained with the nuclear stain Hoechst. The cultures are double labelled with antibodies against glutamine synthetase (Red: Alexa fluor® 555) (B) and SNAP-23 (Green: Alexa fluor® 488) (C). The merged image (D) is an overlay of all channels and this image shows the co-localization of glutamine synthetase and SNAP-23 in cultured rat trigeminal satellite glial cells. Virtually all SGCs seem SNAP-23 positive.

Fluorescence microscopy was used to identify the expression of SNAP-25 and SNAP-23 in SGCs. The SGCs were characterized by double labelling with GS, a specific marker for SGCs in the peripheral ganglia. Figures1 and 2 show, respectively, the immunoreactivity of SNAP-25 and SNAP-23 and how they were distributed. Double labelling between SNAP-25 or SNAP-23 and GS was used to identify positive SGCs for SNAP in cryostat tissue sections of the TG. SNAP-25 and SNAP-23 were also present and observed in cultured cells as presented in Figures3 and 4, respectively.

### Glutamate release evoked by ionomycin

**Figure 5:**
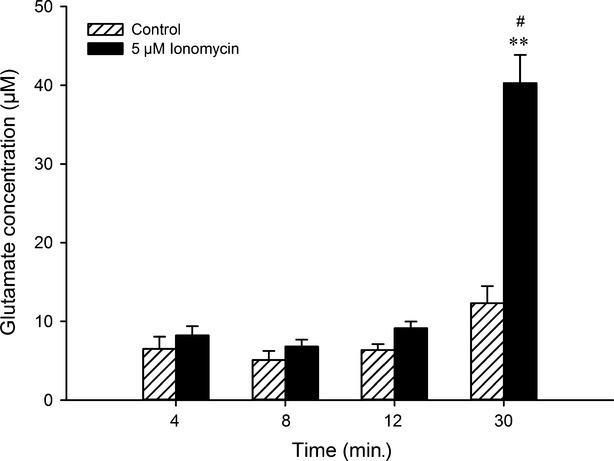
Time-dependent modulation of glutamate release by ionomycin. Glutamate concentration (μM) in culture medium after 4, 8, 12 and 30 min. of 5 μM ionomycin incubation. **P < 0.01 indicates the significant difference of glutamate release when ionomycin is compared with control at 30 min.; ^#^*P* < 0.05 indicates that glutamate release at 30 min is different compared with other stimulation time-points. Results are shown as mean ± SEM.

The time-dependent stimulatory effect of ionomycin on glutamate release was investigated in the cultured SGCs. Samples were obtained at 4, 8, 12 and 30 min. after treatment with ionomycin and control. Ionomycin increased glutamate levels at 30 min., when compared to control (*P* < 0.01). No significant difference was detected between control and the first three time-points (4, 8 and 12 min.) after ionomycin treatment (Fig.5).

### Modulatory effect of BoNTA on ionomycin-evoked release of glutamate from cultured SGCs

**Figure 6:**
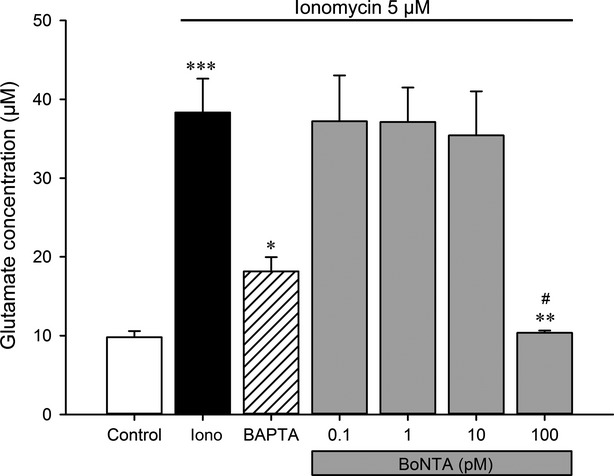
Effect of BoNTA on evoked release of glutamate by ionomycin in 7-day-old primary trigeminal satellite glial cell. The glutamate concentration was determined in the culture medium after treatment with control medium (control), 5 μM ionomycin alone (Iono), 50 μM BAPTA together with 5 μM ionomycin and 0.1, 1, 10 and 100 pM BoNTA together with 5 μM ionomycin. ***P < 0.001 indicates the significant difference between ionomycin-evoked glutamate release compared with control; *P < 0.05 indicates the effectiveness of BAPTA in reversing the ionomycin-evoked glutamate release; **P < 0.01 indicates that 100 pM BoNTA significantly reduced glutamate release in comparison with Iono; ^#^*P* < 0.05 indicates that 100 pM BoNTA significantly reduced glutamate release in comparison with the other three concentrations of BoNTA. Results are shown as mean ± SEM.

The modulatory effect of BoNTA on glutamate release from SGCs was investigated (Fig.6). Stimulation with ionomycin significantly increased the glutamate concentration compared with control (*P* < 0.001). Pre-treatment with 100 pM BoNTA was able to significantly decrease the glutamate concentration when compared to ionomycin alone (*P* < 0.01) or a lower concentration of BoNTA (10 pM, *P* < 0.05). No significant difference was detected for pre-treatment with 0.1, 1 or 10 pM of BoNTA when compared to ionomycin alone (*P* > 0.05). BAPTA significantly decreased glutamate concentration when compared with ionomycin alone (*P* < 0.05).

## Discussion

This study demonstrated that: (*i*) The cell membrane docking proteins SNAP-25 and SNAP-23 are expressed by trigeminal SGCs; (*ii*) cultured SGCs, when stimulated with ionomycin, release glutamate and (*iii*) BoNTA inhibited the release of glutamate from SGCs possibly through cleavage of SNAP-25 and/or SNAP-23, which would inhibit vesicular docking.

### Presence of SNAP-25 and SNAP-23 in trigeminal SGCs

The SNARE (soluble *N*-ethylmaleimide sensitive factor attachment protein receptor) complex comprises a large group of membrane-associated proteins that enables transmitter vesicle docking and its fusion to the cell membrane. The vesicles in response to calcium influx undergo rapid exocytotic fusion and release neurotransmitters such as glutamate 23,24. SNAP-25 is one of the proteins from the SNARE complex, and it is well-known that BoNTA cleaves SNAP-25 and blocks the release of transmitters that are stored in vesicles 18,25. Murine SNAP-23 is also cleaved by BoNTA 26. SNAP-23 is an SNAP-25 analogue protein found in non-neuronal cells 13. In amino acid composition, SNAP-23 is 59% identical to SNAP-25 27. In non-neuronal cells, SNAP-23 performs similar functions in vesicle docking and fusion to membrane for transmitter release 27,28. BoNTA is also known to cleave SNAPs in non-neuronal cells of the central nervous system, such as astrocytes and microglia 13,14. Although it has been shown that SNAP-25 is present in cell bodies of neurons isolated from TG 4, the present study show that both SNAP-25 and SNAP-23 are present in trigeminal SGCs. Interestingly, cultured SGCs maintained the *in vivo* expression of SNAPs. It has been reported that SNAP-25 has been detected at highest level within the first few days after astrocytes culturing, whereas prolonged culture time reduced SNAP-25 dramatically 29; however, several studies from astrocytes and microglia could only detect SNAP-23 13,30. Verderio *et al*. reported the presence of SNAP-23 in Schwann cells 31. Recently, Marinelli *et al*. have provided direct evidence that SNAP-25 is present in astrocytes. In addition, this group has provided direct evidence that cl-SNAP25 is co-localized with GFAP in rat sciatic nerve sections. Considering that GFAP is expressed in non-myelinating cells as well as proliferating Schwann cells, this finding may indirectly demonstrate that BoNTA cleaves SNAP-25 in Schwann cells 32. Further investigations may provide direct evidence; whether inhibitory effect of BoNTA on SGCs as it was seen in our study is a result of interaction with SNAP-25, or SNAP-23 or another unknown mechanism(s). Expression of SNAP-23 or SNAP-25 in glial cells indicates that these cells are capable of calcium-dependent vesicular release of substances, which can be modulated by agents capable of blocking the vesicular release. Trigeminal SGCs contain glutamate, and release it under certain condition such as inflammation or injury 5. This process, if involved in trigeminal nociception, could potentially be modulated to help developing better strategies for treating craniofacial pain conditions.

### Ionomycin-evoked glutamate release

Glutamate is an excitatory neurotransmitter, which plays an essential role in peripheral nociception 33,34. It has been shown that glutamate is one of the key molecules in craniofacial pain conditions including migraine 35. Several preclinical studies have also investigated the role of glutamate in association with nociception and muscle sensitization in rats and in humans 36,37.

Using pharmacological tools, such as KCl, bafilomycin and ionomycin, to elicit glutamate release is commonly used in both neurons and non-neuronal cells. We have recently demonstrated that trigeminal SGCs in rat contain glutamate, express EAAT1 and EAAT2, and, under conditions of elevated potassium concentration, release glutamate through EAATs 11. Here we addressed whether glutamate can be released from trigeminal SGCs by calcium-dependent vesicular exocytosis through the use of the ionophore ionomycin. Ionomycin, has also been used to provoke glutamate release from Schwann cells 15 and central glia, astrocytes 14, at a similar concentration as applied in this study (5 μM).

Our data provide evidence of vesicular release of glutamate by trigeminal SGCs occurs, which can potentially affect TG neurons and neighbouring SGCs that express glutamate receptors 11,38,39. These findings suggest that glutamatergic transmission within TG could impact nociception and neuron-glia cross talk 10. In addition, as it is shown here and elsewhere 40, that cultured SGCs are capable of maintaining some of their functionality for stimulation and release of substances, such as glutamate in culture and hence *in vitro* studies can provide a platform suitable for investigating responsiveness of SGCs to a given compound under different treatment conditions 40.

Mechanisms of glutamate release from astrocytes have already been studied extensively and it has been reported that astrocytes release glutamate that can influence the activity of their neighbouring neurons through six different mechanisms 41, one of those is calcium-dependent exocytosis. However, it is not yet known which pathways and the extent to which each mechanism contributes to the release of glutamate by astrocytes. It is also unclear if similar or different glutamate release mechanisms occur under physiological and pathophysiological conditions. Given the general consideration that SGCs function in the peripheral nervous system is closer to central astrocytes, compared with other types of glial cells 7, it can be postulated that similar mechanisms for glutamate release from SGCs could eventually exist. The calcium-dependent glutamate release from astrocytes suggests exocytosis as a possible mechanism, which utilizes SNARE complex for vesicle fusion 42–44. Both SNAP-23 and SNAP-25 have been found in astrocytes 30; the present study confirmed the existence of SNAP-23 and SNAP-25 in SGCs both *in vivo* and *in vitro*. Isoforms of vesicular glutamate transporters 1, 2 and 3, have also been detected in astrocytes and play a functional role in mediating calcium-dependent glutamate release from astrocytes. We have also found vesicular glutamate transporters 1 and 2 in trigeminal SGCs and have studied functional role of these transporters in glutamate release 11. Our present data advanced understanding of biological role(s) of SNAPs expression on SGCs and calcium-dependent glutamate release from these cells.

### BoNTA reduced ionomycin-evoked glutamate release from SGCs

Consistent with our recent investigations towards elucidating potential roles of SGCs in craniofacial nociception 11,38,40, we addressed here the open question of vesicular release of glutamate by trigeminal SGCs and proposed that if these cells contain glutamate, and release it under specific conditions, what could be a potential mechanism(s). Our results demonstrated that BoNTA reduced glutamate release from trigeminal SGCs, where its effect was concentration-dependent. Although still under further investigation in our lab and by others, based on several *in vivo* and *in vitro* experiments, it is assumed that antinociceptive mechanism of BoNTA is because of cleavage of SNAP-25, one of the SNARE proteins essential for neurotransmitter release 16,45. This protein is located on the cell membrane of neurons and its deactivation is calcium-dependent and prevents the release of several known neurotransmitters contributing in nociception *e.g*. glutamate, Calcitonin gene-related peptide (CGRP), and Substance P (SP) 21,46,47. Since we demonstrated here that trigeminal SGCs express SNAPs, both 25 and 23, *in vivo* and *in vitro*, we proposed that the inhibitory effect of BoNTA on ionomycin-evoked glutamate release seen in the preset study is because of the cleavage of SNAPs by the toxin 26.

It has been proposed that BoNTA can be transported to sensory ganglia and block the release of substances involved in pain transmission from the ganglion neurons 48,49. Recently, a group of researchers have demonstrated the presence of the cleaved SNAP-25 in dorsal root ganglia after BoNTA injection in the periphery 32. This group has also reported that, in the spinal cord, BoNTA may be transcytosed from nociceptive fibres and enter into astrocytes, as shown by the detection of cleaved SNAP-25 in astrocytes. However, the functional consequences of such observations remain largely unknown and require further investigation. For instance, it is still not known whether BoNTA molecule or cleaved SNAP-25 can reach to the TG through this retrograde transport after intramuscular injection into the craniofacial muscles, and if it is so, what would be the consequences on SGCs enveloping the somata of the trigeminal neurons. Further morphological and experimental studies are required to extensively evaluate the potential migration and effects of BoNTA in sensory ganglia following its peripheral administration. This will assist a better understanding of the toxin effects in the peripheral nervous system following treatments for inflammatory or neuropathic pain conditions. Investigating the effect of BoNTA in sensory ganglia must also address potential safety issues.

It is known that BoNTA recognizes and enters neurons by binding to the synaptic vesicle protein SV2 50 but other isoforms in cultured astrocytes 51. Whether the same process occurs when BoNTA gets in contact with SGCs is not known. The assumption is that as vesicle fusion is mediated by a set of SNARE proteins and the light chain of BoNTA exerts a proteolytic function to cleave specific peptide bonds present in the synaptic fusion complex, it can prevent exocytosis of neurotransmitter containing vesicles similar to what has been shown at nerve terminals 50,52. Inhibition of glutamate release from SGCs, shown in this study, emphasizes the potential role of peripheral non-neuronal cells in nociceptive signalling at the level of sensory ganglia.

Our study is not an exemption from limitations and we recognize the value and importance of quantitative information on expression of the SNAPs in trigeminal SGCs. Further investigation to provide this information and also to examine the extent of SNAPs that undergoes cleavage by BoNTA is warranted. Such information might assist in better describing the antinociceptive/analgesic effects of BoNTA at the level of sensory ganglia. Currently, it is less obvious whether the main form of SNAP in SGCs is SNAP-23 or SNAP-25, nor it is determined yet that under which conditions BoNTA cleaves different SNAPs. For example, Vaidyanathan *et al*. 26, demonstrated that BoNTA cleaves murine SNAP-23; however, only at high concentration and after a long incubation time. It is difficult to speculate if our present experiments have achieved similar cleavages as Vaidyanathan *et al*. (3.6%) especially considering the different glial cell types, use of complete form of the toxin instead of L-chain and also different incubation timing. It also needs to be clarified whether SGCs, under different age stage and conditions, would respond differently to BoNTA.

There might be an effect of ‘all or nothing’ in response to the toxin. We have presented a dose–response result in this study; however, it has been shown that for example, at 3 U/kg, BoNTA seems not to affect the carrageenan- and capsaicin-evoked pain, but at slightly higher dose (3.5 U/kg), it does. Interestingly, increasing the dose to 5 and 7 U/kg, BoNTA exerts similar analgesic activity 53. Similar antinociceptive effect of 3.5, 7 and 15 U/kg BoNTA doses was reported in a formalin test 47. Increased analgesic effects have been shown to occur at higher doses of the applied toxin (20–40 U/kg) but animal motor performance is affected in high doses and most likely interfere with the sensory responsiveness 47,54. Consistent with basic research results, it seems that clinical trials have not addressed a clear dose–response of BoNTA and the employed doses are mainly based on the clinical effects 55. These observations might be proven for the effect of BoNTA on glial cells. There might be a lowest potential dose of BoNTA in which the SGCs respond and further increase would potentially not add any additional increase in responsiveness. We are not able to fully clarify this point with the current results, but as we have observed that the inhibitory effect of BoNTA on glutamate release occurred between 10 and 100 pM, it would be useful to see what would be the lowest effective concentration of BoNTA between 10 and 100 pM.

## Conclusions

Results from this study demonstrated that SNAP-25 and SNAP-23 are present in trigeminal SGCs. It was also demonstrated that SGCs, when stimulated with ionomycin, release glutamate, which is inhibited by BoNTA. This study also shed lights into analgesic action of BoNTA at the level of sensory ganglia, with an emphasis on the role of peripheral non-neuronal cells.

## Footnotes

* Worzella T, Niles A, Helley L, Conley M and Riss T. An Image-Based Method for Real-Time Monitoring of Cytotoxicity Using CellTox™ Green Dye and the Essen Bioscience IncuCyte™ FLR Live Content Imaging System. Promega Corporation: http://www.promega.com/resources/pubhub/monitoring-cytotoxicity-using-celltox-green-and-the-incucyte-live-content-imagingsystem/Updated October 2013.
